# Editorial Announcements

**Published:** 1913-03

**Authors:** 


### Editorial Announcements

Article by Our Associate Editor For Germany. Dr. C. A. Ewald has contributed a valuable article on Parenchymatous Gastric Haemorrhages, which has already been translated and will be published in the April or May issue.

---

Lack of Credit For News Items. It has been found impracticable to give credit for news items of various kinds, especially as these are often found in newspapers and exchanges which have obviously obtained them from other sources. We wish, however, to express our indebtedness to the *Journal of the A. M. A.* for a large minority of obituary items, chiefly of physicians formerly but not at present residents of Western New York, and to state that the *Boston Med. and Surg. Journal* is especially active in collecting notes of longevity, etc. Items regarding professional societies are also, to a large degree, contributed by the secretaries. We feel free to appropriate news items from any available source and do not expect credit to this Journal for any similar item nor for abstracts, which should be designated according to the original source.

